# Comparison of detection methods and genome quality when quantifying nuclear mitochondrial insertions in vertebrate genomes

**DOI:** 10.3389/fgene.2022.984513

**Published:** 2022-11-22

**Authors:** Deborah A. Triant, William R. Pearson

**Affiliations:** Department of Biochemistry and Molecular Genetics, University of Virginia, Charlottesville, VA, United States

**Keywords:** BLASTN, karyotypic diversity, *Microtus*, NUMT, rodents, scoring matrix, TFASTX

## Abstract

The integration of mitochondrial genome fragments into the nuclear genome is well documented, and the transfer of these mitochondrial nuclear pseudogenes (numts) is thought to be an ongoing evolutionary process. With the increasing number of eukaryotic genomes available, genome-wide distributions of numts are often surveyed. However, inconsistencies in genome quality can reduce the accuracy of numt estimates, and methods used for identification can be complicated by the diverse sizes and ages of numts. Numts have been previously characterized in rodent genomes and it was postulated that they might be more prevalent in a group of voles with rapidly evolving karyotypes. Here, we examine 37 rodent genomes, and an additional 26 vertebrate genomes, while also considering numt detection methods. We identify numts using DNA:DNA and protein:translated-DNA similarity searches and compare numt distributions among rodent and vertebrate taxa to assess whether some groups are more susceptible to transfer. A combination of protein sequence comparisons (protein:translated-DNA) and BLASTN genomic DNA searches detect 50% more numts than genomic DNA:DNA searches alone. In addition, higher-quality RefSeq genomes produce lower estimates of numts than GenBank genomes, suggesting that lower quality genome assemblies can overestimate numts abundance. Phylogenetic analysis shows that mitochondrial transfers are not associated with karyotypic diversity among rodents. Surprisingly, we did not find a strong correlation between numt counts and genome size. Estimates using DNA: DNA analyses can underestimate the amount of mitochondrial DNA that is transferred to the nucleus.

## Introduction

Mitochondrial and nuclear genomes have been co-evolving for more than a billion years, so that most of the proteins needed for mitochondrial function are now found in the nuclear genome following the gradual transfer of ancestral mitochondrial (mtDNA) genes to the nucleus (du Buy and Riley, 1967; Lang et al., 1999). The transfer and insertion of mtDNA genome fragments into the nuclear genome has continued, with nuclear copies of mtDNA fragments documented in a variety of species (Hazkani-Covo et al., 2010; Yan et al., 2019; Ding et al., 2021; Zhang et al., 2022). These nuclear insertions or “numts” (Lopez et al., 1994) are widespread among eukaryotes, but despite their ubiquity, numt function is largely unknown. Once integrated into the nuclear genome, numts are no longer under the evolutionary constraints of the mtDNA genome and can evolve as noncoding nuclear sequences that can fragment after insertion (Hazkani-Covo et al., 2003; Kim et al., 2006). The accumulation of mutations in older numt insertions can alter the transferred sequence so that it is no longer perceived as a mtDNA fragment. When numts are co-amplified with mtDNA during PCR-based studies or misaligned during similarity searches, they can compromise studies involving heteroplasmy (Maude et al., 2019), DNA barcoding (Song et al., 2008), ancient DNA (Ovchinnikov and Kholina, 2010) and phylogenetics (Lucas et al., 2022). Numts have also incorrectly supported the bi-parental inheritance of mtDNA (Luo et al., 2018).

With the increasing number of nuclear genome sequences available, many genome-wide assessments of numts have been described (Pereira and Baker, 2004; Calabrese et al., 2012; Nacer and Raposo do Amaral, 2017; Liang et al., 2018). However, differences in genome assembly quality can reduce the accuracy of numt transfer estimates, especially when sequence alignment methods are used (Tsuji et al., 2012; Grau et al., 2020). The diversity in the age and size of numts can also complicate numt estimates as numts can be duplicated within the genome post-insertion and can vary in length from short fragments to numts that span the entire mtDNA genome. Correlations between genome size and numt abundance have been suggested and patterns of numt organization tend to vary across species (reviewed in Puertas and González-Sánchez, 2020).

Numts are typically identified by comparing the complete mitochondrial genome sequence to the nuclear genome sequence, using the BLASTN similarity search program (Camacho et al., 2009) to compare the mitochondrial DNA sequence to a target genome [e.g., (Pamilo et al., 2007; Féménia et al., 2021)]. However, similarity searches done with protein:translated-DNA are more sensitive than DNA:DNA searches, because their scores are calculated using protein similarity scoring matrices (Pearson et al., 1997; Pearson, 2019). Protein:translated-DNA searches routinely allow evolutionary look back times that are 5–10-fold longer than DNA:DNA alignments, so we examined the effect of these more sensitive search methods on numt estimates.

How numts integrate into the nuclear genome is unclear. One hypothesis proposes that mtDNA is introduced at nuclear double-strand DNA breaks by non-homologous end-joining repair machinery (Blanchard and Schmidt, 1996; Ricchetti et al., 2004; Hazkani-Covo and Covo, 2008). DNA strand breaks are required for chromosomal structural changes, such as inversions and translocations, and these rearrangements can influence chromosomal evolution (Garagna et al., 2001; Dobigny et al., 2017), suggesting a possible relationship between numt quantities and karyotypic diversity. Estimations of numts in rodents have linked high rates of karyotypic evolution with the accumulation of numts (Triant and DeWoody, 2007a; Triant and DeWoody, 2008). The rodent subfamily Arvicolinae (Cricetidae) consists of ∼150 species of voles and lemmings with almost half of the species within the genus *Microtus* (Musser and Carleton, 2005; Galewski et al., 2006). Rates of speciation and karyotypic evolution among *Microtus* voles are among the fastest known for mammals (Modi, 1987; Fabre et al., 2012; Steppan and Schenk, 2017) with karyotypes characterized by numerous chromosomal rearrangements (Lemskaya et al., 2010; Romanenko et al., 2018). If the insertion of mtDNA is driven by chromosomal repair mechanisms, then perhaps the chromosomal structural rearrangements that have occurred throughout voles’ evolutionary history is facilitating this process leading to a high numt content. Thus, we were interested in examining the correlation between karyotypic evolution and numt transfer.

In this paper, we examine the relationship between karyotypic diversity in *Microtus* voles and numt transfer using a diverse set of rodent genomes. To better estimate numt transfers, we tested seven different sequence alignment methods. Previous searches for numts in rodent nuclear genomes used just a few rodents and searches were done with BLASTN (Triant and DeWoody, 2007b). In this work, we expand the number of rodents for which nuclear and mitochondrial genomes are available to compare similarity search algorithms, sequence types (DNA:DNA and protein:translated-DNA), protein scoring matrices and genome assembly quality. We also extend the same search techniques to another dataset of vertebrate genomes that range in size and quality.

## Materials and methods

### Mitochondrial and genomic sequences

**FIGURE 1:**
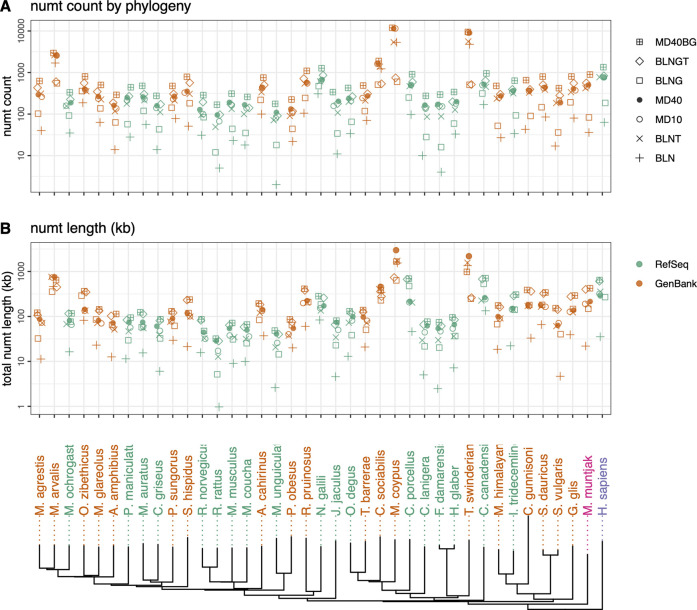
Rodent neighbor-joining tree constructed with full mtDNA genome sequences with *M. muntjak* colored in pink and *H. sapiens* colored in purple. GenBank genomes are colored orange and RefSeq colored green. The three vole species from the genus *Microtus* (*M. agrestis, M. arvalis* and *M. ochrogaster*) are found on the far left. Total numt counts **(A)** and length **(B)** are shown for each species for the 7 different similarity searches run are shown as follows: 1) plus sign (+): coding sequence queries—BLASTN DNA:DNA with “-task megablast” default option (BLN); 2) (x): coding sequence queries—BLASTN DNA:DNA with “-task blastn” option (BLNT); 3) open circle: coding sequence queries—TFASTX protein:translated-DNA searches with the MD10 protein scoring matrix (MD10); 4) filled circle: coding sequence queries—TFASTX protein:translated-DNA searches with the MD40 protein scoring matrix (MD40); 5) open square: whole mtDNA genome queries—BLASTN DNA:DNA with “-task megablast” default (BLNG); 6) diamond: whole mtDNA genome queries—BLASTN DNA:DNA with “-task blastn” option (BLNGT); 7) square with plus inside: combined numts found only with coding sequence queries TFASTX protein:translated-DNA with the MD40 scoring matrix, only those found with whole mtDNA genomes—BLASTN DNA:DNA that includes numts from non-coding portions of the genome and overlapping numts found with both (MD40BG).

Mitochondrial coding sequences (both protein and nucleotide) and complete mitochondrial genome sequences were downloaded from the National Center for Biotechnology (NCBI), as were complete nuclear (or nuclear plus mitochondrial) genome assemblies. If a nuclear genome assembly was missing the mitochondrial genome, we downloaded it separately. We selected 37-rodent species, for which either GenBank or RefSeq genome assemblies were available (referred to as the “rodent” dataset; Supplementary Table S1). We also included the assembly for human because of its detailed annotation and the assembly for muntjak, an Asian deer that exhibits rapid karyotypic evolution among mammals (Wurster and Benirschke, 1967, 1970; Mudd et al., 2020), for a total of 39 genomes (20 from GenBank and 19 from RefSeq; Figure 1 and Supplementary Table S1). There was no annotated mtDNA genome for *Cynomys gunnisoni* so the mtDNA genome for the subspecies *C. gunnisoni gunnisoni* was used as the query sequence. MtDNA coding sequences (both protein and nucleotide) were also downloaded for each species from the same annotated mtDNA genome sequence as either the “FASTA nucleotides” or “FASTA proteins” (Supplementary Table S2).

A second vertebrate dataset was examined to explore the relationship between numt transfer and genome size over a longer evolutionary timescale. We sought to sample from a range of genome sizes using the better-annotated RefSeq assemblies when available. Human, mouse, and rat genomes were used in both genome sets. The vertebrate dataset consisted of 21 RefSeq and 6 GenBank genome assemblies, including the 3 RefSeq assemblies for *H. sapiens, M. musculus* and *R. norvegicus* that were also used in the rodent genome set (Supplementary Table S1). Genome sizes ranged from 0.38–34.56 GB. MtDNA coding sequences were downloaded as described for the rodent genome set. We sampled across a range of genome sizes within each taxonomic group from the smallest fish genome to the largest lungfish and salamander genomes. When a group had a relatively consistent range of genome sizes, we sampled from the genomes that were of good quality (e.g., for the consistent bird genomes, we used the chicken and zebra finch genomes). We also sampled from the marsupials as this group was reported to have an abundance of numts in their nuclear genomes relative to other mammals (Hazkani-Covo, 2022). A complete list of species and accession numbers with versions for the sequences used is provided in Supplementary Table S1 (species, mitochondrial genome accession, genome assembly accession) and Supplementary Table S2 (mitochondrial protein-coding sequence accessions).

### Sequence searches

Similarity searches with mitochondrial sequences were run against the nuclear genomes using three sets of query sequences per species: 1) the individual protein sequences of the 13 mtDNA protein-coding genes were compared to the nuclear genome using TFASTX (version 36.3.8i, September 2021; (Pearson et al., 1997), 2) the nucleotide sequences of the 13 mtDNA protein-coding genes were compared to the nuclear genome using BLASTN (version 2.12.0+; Camacho et al., 2009), and 3) the entire mitochondrial genome nucleotide sequence was compared to the nuclear genome with BLASTN. Searches using DNA vs. DNA for both mtDNA protein-coding genes and entire mtDNA genomes as search queries were performed with BLASTN with two different algorithm options, described below. Searches using protein:translated-DNA were performed with TFASTX with two different scoring matrices. Because the genetic codes between nuclear and mtDNA differ, we used the mtDNA translation table for alignments. TFASTX searches used the command line output option ‘‘-m8CBl”, which produces the same output format as BLASTN with the “-outfmt '7 qseqid qlen sseqid slen pident length mismatch gapopen qstart qend sstart send evalue bitscore score btop' command line output option. Search results were loaded into a mySQL database (Pearson and Mackey, 2017) for further analysis.

BLASTN searches were conducted using the default parameters, no -task option, which defaults to “-task megablast” (labeled in the Figures as “BLN”), or with the ‘‘-task blastn” option (labeled as “BLNT”), which produces a more sensitive search. Two types of scoring matrices were evaluated with the TFASTX searches: the MDM10 scoring matrix using the ‘‘-s MD10’’ option (labeled as “MD10”) and the MDM40 scoring matrix using the ‘‘-s MD40’’ option (labeled as “MD40”) (Jones et al., 1992). The MD10 matrix targets alignments that are 90% identical, while the MD40 matrix targets alignments that are about 65% identical (Pearson, 2013b).

### Estimating numt counts

Numt alignments were counted if they were at least 30 nucleotides long (or 10 amino acids) and had a statistical significance (E()-value) of 0.001. We used a threshold of 30 nucleotides (10 amino acids) so that the alignments would be long enough to generate a statistically significant score. Full-length exact-matches (> 99% identity) alignments were excluded to remove self-hits to authentic mitochondrial sequences. Because searching with the 13 protein-coding genes can overestimate the number of numts by breaking long multi-gene nuclear insertions into individual gene alignments, searches with protein-coding regions were post-processed to combine adjacent multi-gene alignments into a single longer alignment. To look at numt transfer across the mitochondrial genome, genomic search alignments with the “-task blastn” option (“BLNGT”) were mapped back to the mitochondrial genome in 16 non-overlapping fragments and the median number of numts across the 39 “rodent” genomes in each interval was calculated. To account for any possible false positives, we determined the fraction of numts that would also be found with more stringent E()-values, $<10^{-6}$ for TFASTX and $<10^{-10}$ for BLASTN, (Supplementary Table S1).

Merged estimates of numt counts (labeled in the Figures as “MD40BG”) combined the results of the two most sensitive strategies, TFASTX/MD40 searches with the 13 mitochondrial proteins, and BLASTN “-task blastn” searches with the entire mitochondrial genome. Because the two methods produce overlapping sets of numt alignments, alignments from each of the searches were merged based on nuclear chromosome location using a python script. Merged counts included alignments identified only by TFASTX/MD40 (MD40), only by BLASTN “-task blastn” whole genome (labeled as “BLNGT”), or by both methods. Alignments identified by both methods were counted only once.

### Comparison to an existing human numts database

Human numt locations assembled by Simone et al. (2011) were downloaded from the UCSC genome browser (https://genome.ucsc.edu/cgi-bin/hgTrackUi?db=hg19&g=numtSeq) as a BED file using the UCSC table browser (Kent et al., 2002). Numts were originally mapped onto the hg19 genome assembly but the current study uses build hg38. Therefore, coordinates were converted with the UCSC Liftover tool (https://genome.ucsc.edu/cgi-bin/hgLiftOver). Coordinates from the BLASTN “-task blastn” genome searches (BLNGT) were converted to bed format. Because the NCBI hg38 genome sequence uses non-chromosomal names for accessions, we removed numt references to incompletely assembled or unmapped chromosome locations; only numts on chr1-chr22, chrX, and chrY were compared. UCSC numt coordinates and BLNGT coordinates were compared using the “bedtools intersect” function from Bedtools2 version 2.30.0 (Quinlan and Hall, 2010).

Numt counts were totaled and plotted with ‘R’ scripts using ggplot2 (Wickham, 2016). A rodent phylogeny was constructed from a multiple sequence alignment of the mitochondrial genomes listed in this study using MUSCLE (Edgar, 2004) followed by a distance calculation using Biopython’s (Cock et al., 2009) Bio.Phylo.Treeconstruction DistanceCalculator () and DistanceTreeConstructor () functions to build a Neighbor Joining tree (Saitou and Nei, 1987). Correlations between genome size and numt count were estimated using the ‘R’ cor.test () function. The software and datasets used in this analysis are available from https://github.com/wrpearson/numts2022.

## Results

### Mitochondrial-nuclear transfer is not correlated with karyotypic diversity in rodents

**FIGURE 2:**
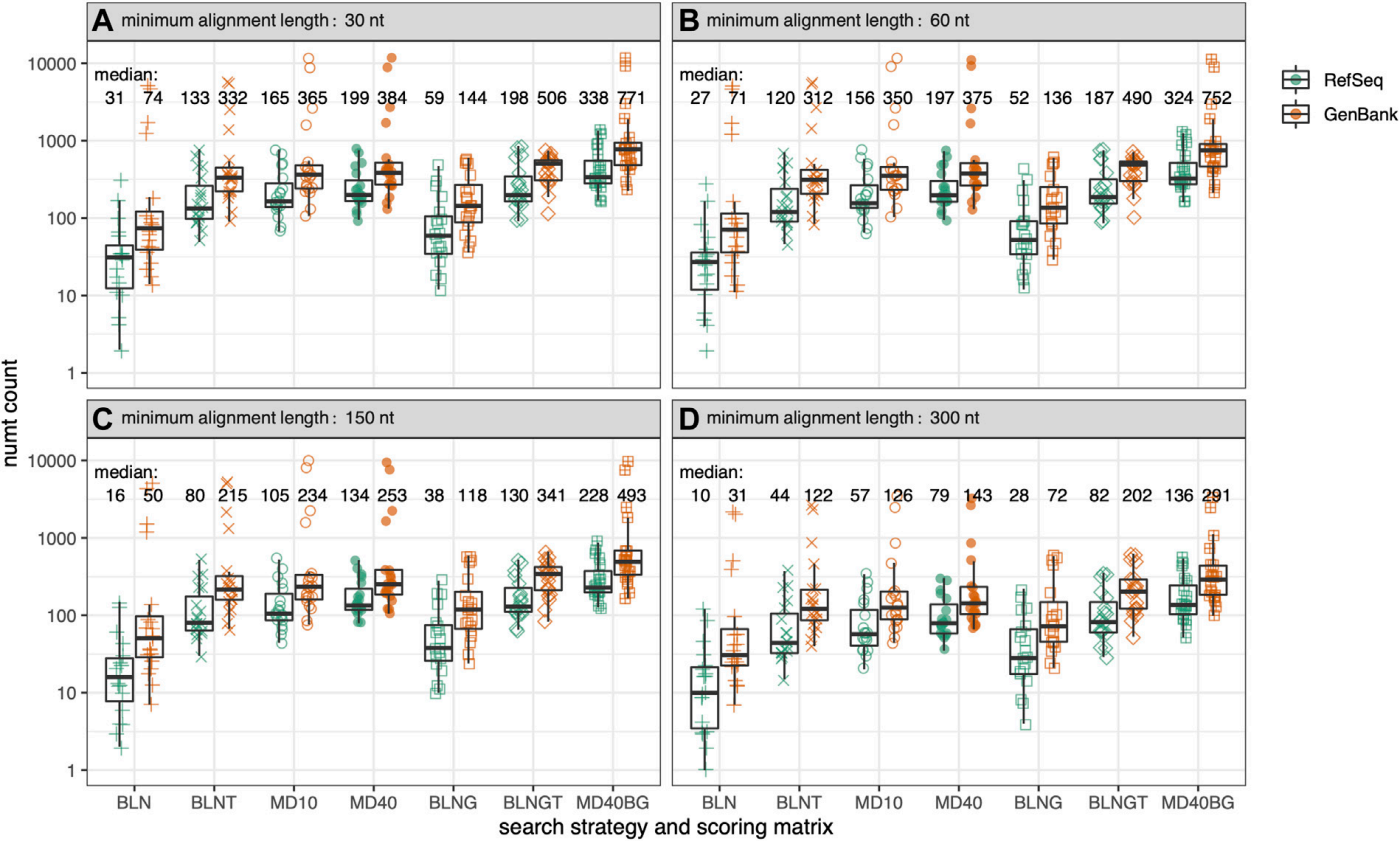
Total numt counts for the genomes in Figure 1 for 7 different similarity searches as a function of alignment length. Within each box plot, there are 39-symbols for the 39-genomes displayed in Figure 1 color-coded by genome type. **(A)** alignments at least 30 nt/10 aa; **(B)** at least 60 nt/20 aa; **(C)** at least 150 nt/50 aa; **(D)** at least 300 nt/100 aa. Median values are given for each type of similarity search with GenBank mtDNA genomes colored in orange and RefSeq mtDNA genomes colored in green. The symbols for the 7 different similarity searches run are the same as in Figure 1, and are labeled on the x-axis.

We compared voles in the genus *Microtus* (*M*. *agrestis*, *M*. *arvalis*, and *M*. *ochrogaster*), which have undergone rapid karyotypic evolution, with 34 other rodent genomes, as well as human and the muntjak deer (20 genomes from GenBank and 19 from RefSeq (Figures 1, 2; Supplementary Tables S1, S2). The number (Figure 1A) and length (Figure 1B) of mitochondrial/nuclear transfer events in the rodent genome set are plotted on a mtDNA genome phylogeny (human was included for comparison of a well-annotated genome and *M. muntjak* because of its chromosomal diversity among mammals). For each organism, transfers were measured with seven different types of similarity search strategies: four using BLASTN (mitochondrial DNA:nuclear genomic DNA searches), two using TFASTX (mitochondrial protein *versus* translated nuclear genome DNA) with either the MD40 protein scoring matrix or the MD10 protein scoring matrix, and one method that combined the most sensitive BLASTN search with the most sensitive TFASTX search (MD40BG). Three sets of sequences were used for similarity searches: 1) the individual protein sequences of the 13 mtDNA protein-coding genes, 2) the nucleotide sequences of the 13 mtDNA protein-coding genes, and 3) the entire mtDNA genome nucleotide sequence. The different search strategies, and the number of numts they detect, are summarized in Table 1.

**TABLE 1:** Similarity searching strategies used in this study: program, query sequences, scoring matrix, abbreviations used within the text, the number of numts found in mouse, rat, human and the medians for rodent Refseq and GenBank genomes.

Program	Query	Scoring	Abbreviation	Mouse	Rat	Human	RefSeq	GenBank
BLASTN	mtCDS	-task megablast	BLN	23	31	63	31	74
BLASTN	mtCDS	-task blastn	BLNT	133	95	772	133	332
TFASTX	Mt-proteins	-s MD10	MD10	165	100	765	165	365
TFASTX	Mt-proteins	-s MD40	MD40	191	128	758	199	384
BLASTN	mtGenome	-task megablasst	BLNG	44	84	185	59	144
BLASTN	mtGenome	-task blastn	BLNGT	171	194	834	198	506
BLASTN + TFX	mtGenome + proteins	Blastn + MD40	MD40BG	310	286	1,348	338	771

Numt transfer can be quantified in two ways, either by considering the number of mitochondrial to nuclear transfer events (“numt counts”, Figure 1A), or by calculating the total amount of mtDNA sequence that was transferred to the nucleus (“numt length”, Figure 1B). As Figures 1A,B show, numt count and numt length show virtually identical trends. The same similarity (counts vs length) can be seen when looking at vertebrate numt transfer (Figure 5). Because both measures of transfer show similar trends, we have used numt count to investigate the effects of search similarity, genome origin, and genome size as reported below. We also report the median numt lengths and longest numt found for each species in Supplementary Table S1.

Among the rodent genomes, the median number of numts calculated using the most sensitive MD40BG method ranges from 168 to 11,930 (median 477); 25% of the genomes have fewer than 310 numts, and 25% have more than 803 (Figure 1; Supplementary Table S1). The *Microtus* voles are not unusual, with 625 (*M. agrestis*), 334 (*M. ochrogaster*), and 2,952 (*M. arvalis*) numts (the *M. arvalis* number is probably inflated by a poor genome assembly, as discussed below). Thus, despite their unusually high level of karyotypic diversity, the range of numts found in *Microtus* vole nuclear genomes is typical for rodents. Likewise, the *M. muntjak* genome, which has also seen considerable chromosomal rearrangement, is just above the third quartile for numts (895). We do not see a strong association between karyotypic diversity and mitochondrial-nuclear transfer in either the *Microtus* voles or *M. muntjak*. Two Arvicoline voles, which are not karyotypically diverse, have numt counts similar to the *Microtus* voles: 290 (*A. amphibius*) and 502 (*M. glareolus*).

### Different search strategies identify different sets of numts

As the range of symbols for each organism in Figure 1 shows, different search strategies detect different numbers of numts. The sensitivities of different search strategies are illustrated in more detail in Figure 2, which reports the number of numts identified from either RefSeq or GenBank genomes. In Figure 2, each species from Figure 1 is represented within a box plot color-coded by genome type. To better display the sensitivities of the different methods at different alignment lengths, we consider alignments with minimum lengths of 30, 60, 150, and 300 nucleotides (nt) (10, 20, 50, or 100 amino acids for TFASTX searches).

As Figures 1, 2; Table 1 show, BLASTN using the default “-task megablast” option is the least sensitive search. The BLASTN default typically identifies about one-quarter as many numts as either BLASTN in its more sensitive mode (“-task blastn”), or the TFASTX searches. For RefSeq genomes and alignments ≥ 30 nt, BLASTN (default, BLN) the median number of numts identified across the 39 genomes is 31, compared with 133 (BLASTN “-task blastn”, BLNT). For alignments ≥ 300 nt (Figure 2), the median number of numts drops to 10 (BLN) vs. 44 (BLNT). BLNT is slightly less sensitive than TFASTX with the MD10 scoring matrix (133 vs 165 median numts at ≥ 30 nt; 44 vs. 57 at ≥ 300 nt) while BLNT is about 67% as sensitive as TFASTX with MD40. Similar improvements in sensitivity are seen when looking at median numt count in 21 vertebrate RefSeq genomes: 10 vs. 52 for BLN vs. BLNT at ≥ 30 nt, 3 vs. 15 at ≥ 300 nt; 52 BLNT vs. 71 TFASTX/MD40 at ≥ 30 nt, but 15 vs. 17 at ≥ 300 nt. (Supplementary Figure S1).

While protein:translated-DNA (TFASTX) searches are more sensitive than the most sensitive BLASTN searches, searches with individual coding sequences miss the non-protein-coding portions of the mitochondrial genome. BLNG and BLNGT (“-task blastn”) search with the entire mitochondrial genome, the traditional strategy for identifying numts. These searches identify as many numts as TFASTX/MD40, and sometimes more (for RefSeq genomes, medians of 198 BLNGT vs 199 TFASTX/MD40 at ≥ 30 nt; 82 vs. 79 at ≥ 300 nt, Figure 2). In the vertebrate RefSeq genome set, BLNGT found more numts than TFASTX/MD40, with medians of 101 (BLNGT) vs. 71 (TFASTX/MD40) at ≥ 30 nt and 38 vs. 17 at ≥ 300 nt (Supplementary Figure S1).

**FIGURE 3:**
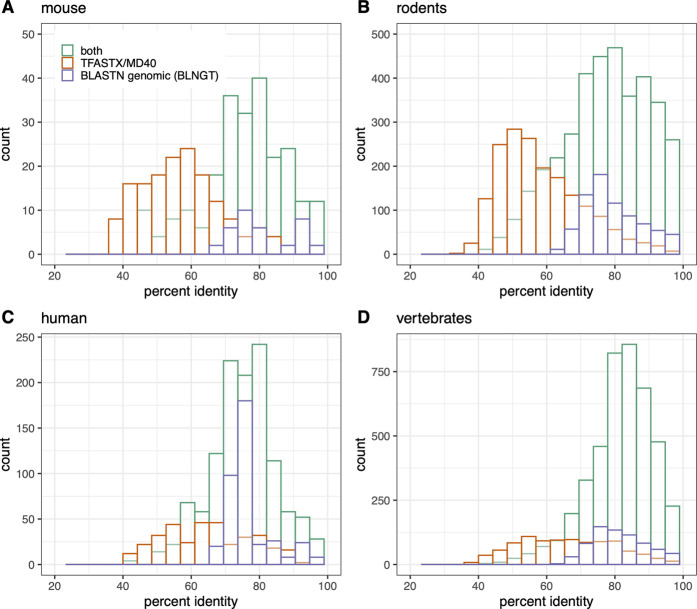
Numt counts (MD40BG) vs percent identity separated by TFASTX/MD40 only (orange), BLASTN “-task blastn” whole mitochondrial genome only (BLNGT, purple), or both methods (green). **(A)** *Mus musculus*; **(B)** rodent RefSeq genomes used in Figure 1; **(C)** *Homo sapiens*; **(D)** vertebrate RefSeq genomes. The percent identities are compared in each panel for 1) TFASTX (orange): numts found only with coding sequence queries TFASTX protein:translated-DNA with the MD40 scoring matrix; 2) BLASTN (purple): numts found only with whole mtDNA genomes - BLASTN DNA:DNA with “-task blastn” option that includes numts from non-coding portions of the genome; 3) numts found with both methods (TFASTX and BLASTN, green).

While TFASTX/MD40 and BLNGT find similar numbers of numts, many of the numt locations are different. Because it searches with the entire genome, BLNGT finds numts corresponding to the non-protein-coding portion of the mitochondrial genome (about 33%) that cannot be detected with TFASTX. However, TFASTX searches are more sensitive than BLASTN “-task blastn”, allowing additional numts to be found from the protein-coding portion (66%) of the mitochondrial genome. To produce a comprehensive estimate of mitochondrial transfer, we combined the results from TFASTX/MD40 and BLNGT by examining the nuclear genome alignment coordinates and recording whether the alignment was found by TFASTX/MD40, BLNGT, or both search methods. The merged alignment results are shown as MD40BG in Figures 1, 2 and Supplementary Figures S1, S2, S4. Because we account for numt alignments found by both methods, the merged numt count is not the sum of the MD40 and BLNGT counts (Figure 3).

We evaluated the accuracy of our BLNGT chromosome locations in humans by comparing their coordinates to those of Simone et al., (2011) downloaded from the UCSC browser. Of the 755 numt sites on chr1-22,X and Y from the UCSC browser, 702 (93%) were found overlapping our BLNGT (blastn “-task blastn” with the entire mitochondrial genome) sites by 90%; 715 (95%) overlapped at 50% coverage. Virtually identical results (702–720 overlapping sites) were found with the most comprehensive MD40BG approach, suggesting that the most sensitive approach finds most of the previously identified and validated numts in the human genome (Simone et al., 2011).

### Lower quality genome assemblies overestimate numt counts

**FIGURE 4:**
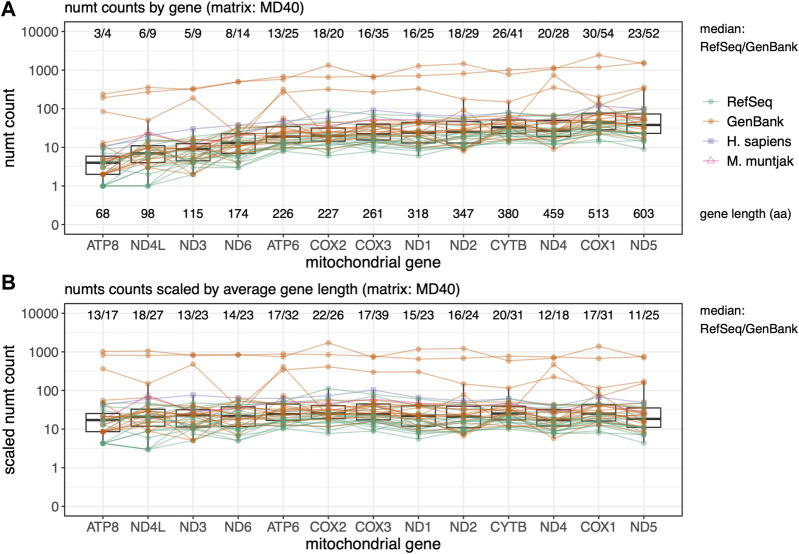
Rodent median numt counts for mtDNA protein genes found using TFASTX with the MD40 scoring matrix. Genes are sorted by length from shortest to longest. GenBank genomes are colored orange and RefSeq colored green with *M. muntjak* colored in pink and *H. sapiens* colored in purple. **(A)** Total counts per gene ordered by gene length in amino acids (aa); **(B)** Numt counts scaled by average gene length. Lines connect numt counts from mtDNA genes in the same species.

**FIGURE 5:**
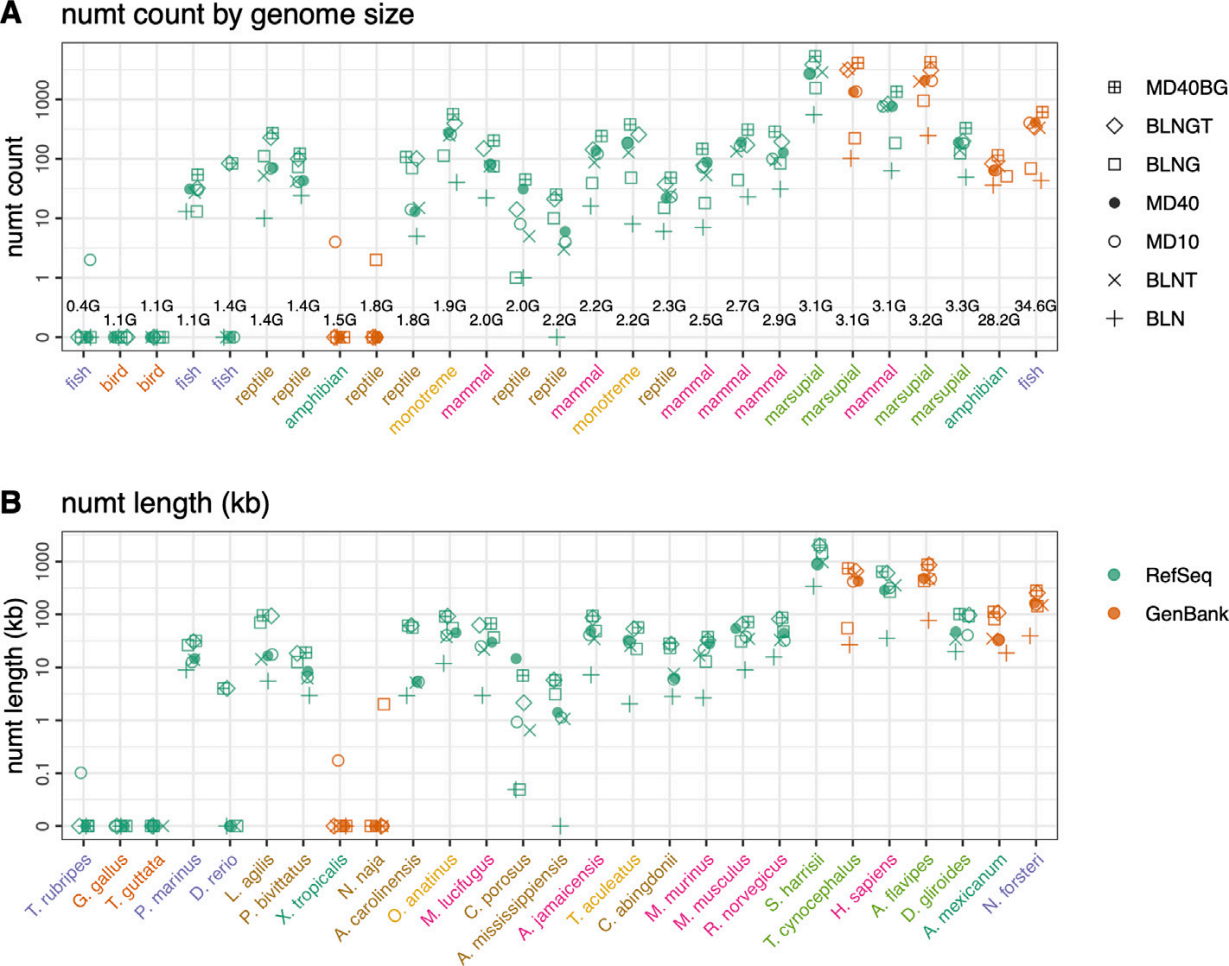
Mitochondrial nuclear transfer across vertebrate genomes. **(A)** Total numt counts and **(B)** total numt length (kb) for the 7 different similarity searches ordered by genome size (GB) from smallest to largest. Genome sizes are listed in the top panel and species are listed on the bottom panel but are consistent for both panels. GenBank mtDNA genomes colored in orange and RefSeq mtDNA genomes colored in green. Scientific names shown in panel B are colored by vertebrate classes shown in panel **(A)**. Numt counts **(A)** and lengths **(B)** for each species for the 7 different similarity searches run are shown as follows: 1) plus sign (+): coding sequence queries—BLASTN DNA:DNA with “-task megablast” default option (BLN); 2) (x): coding sequence queries—BLASTN DNA:DNA with “-task blastn” option (BLNT); 3) open circle: coding sequence queries—TFASTX protein:translated-DNA searches with the MD10 protein scoring matrix (MD10); 4) filled circle: coding sequence queries—TFASTX protein:translated-DNA searches with the MD40 protein scoring matrix (MD40); 5) open square: whole mtDNA genome queries—BLASTN DNA:DNA with “-task megablast” default (BLNG); 6) diamond: whole mtDNA genome queries—BLASTN DNA:DNA with “-task blastn” option (BLNGT); 7) square with plus inside: combined numts found only with coding sequence queries TFASTX protein:translated-DNA with the MD40 scoring matrix, only those found with whole mtDNA genomes—BLASTN DNA:DNA that includes numts from non-coding portions of the genome and overlapping numts found with both (MD40BG).

On average, fewer numts are found in RefSeq genomes than in GenBank genomes in the rodent genome sets (Figures 1, 2, 4) and in the vertebrate genomes (Figure 5). To view the differences in RefSeq and GenBank genome numt counts in more detail, we plotted counts as a function of alignment length in Figure 2. The four species with the highest numt count, regardless of search strategy (the four highest symbols in Figures 2, 4) are *C*. *sociabilis, M*. *arvalis, M*. *coypus* and *T*. *swinderianus*, so we speculated that these four genomes produced most of the discrepancy between the RefSeq and GenBank numt counts. However, when these four genomes are removed from the rodent set, the differences between RefSeq and GenBank numt count medians changes very little (Supplementary Figure S2). This discrepancy between RefSeq and GenBank genome counts is found at different alignment lengths (Figure 2; Supplementary Figure S2), and across each of the mtDNA protein-coding genes (Figure 4). The *M*. *coypus* genome has a large number of contigs (1.6 million) and an N50 of 28 kb. In contrast, the rodent RefSeq genome with the largest number of numts (*N*. *galili*, 1,280 numts) assembles into 356,000 contigs (N50 = 30,353), while a rodent with a more typical number of numts (*M*. *agrestis*, 625 numts) has been assembled into fewer than 7,000 contigs. We included only six GenBank genomes in the vertebrate set because we concluded that RefSeq genome assemblies would provide more accurate numt estimates. Poorly assembled GenBank genomes have many more genome fragments that can dramatically increase the apparent amount of mitochondrial nuclear transfer.

### The mtDNA genome is transferred uniformly across its length

Parts of different protein-coding genes appear in the nuclear genome at different frequencies (Figure 4) - fragments from shorter genes are transferred less frequently, and fragments from longer genes more frequently (Figure 4A). As was seen with the total numt counts and lengths (Figures 1, 2), the GenBank genomes consistently display more mitochondrial transfer, suggesting that lower-quality genome numt counts are likely to be overestimates. When the numt counts are normalized to correct for protein length, the transfer frequencies are relatively uniform (Figure 4B), as would be expected if the mitochondrial genome fragmentation and transfer process were random. To further examine preferential transfer of portions of the mtDNA Genome, we divided the mtDNA genome into 16 non-overlapping segments and plotted them by genome interval with the median number of numts across the 39 species in the rodent set found at each interval with the BLASTN “-task blastn” method (Supplementary Figure S3). This unbiased measure of mitochondrial genome transfer does not show any locally strong transfer preferences across the mitochondrial genome.

### Numt correlation with genome size

In addition to examining the relationship between karyotypic diversity and mtDNA-nuclear transfer by comparing voles with other rodents, we examined the relationship between genome size and numt transfer with a more diverse vertebrate genome set (Figure 5; Supplementary Table S1, and Supplementary Figure S4). The much larger range of vertebrate genome sizes (from 0.4 to 35 GB), together with the longer vertebrate timescale, 400 MYA vs. 60 MYA for rodents (Benton and Donoghue, 2006; dos Reis et al., 2015) allow us to explore the relationship between mtDNA transfer and genome size. In selecting vertebrates for this analysis, we sought groups of more closely related organisms with contrasting genome sizes, e.g., small and large genome amphibians (*X. tropicalis*, genome size 1.4 GB vs. *A. mexicanum* 28.2 GB) and fish (*T. rubripes*, genome size 0.38 GB vs. *D. rerio* 1.4 GB and the ancestral lung fish, *N. forsteri*, genome size 34.6 GB). We also sampled five organisms that have been reported to have high number of numts, the platypus, *O. anatinus*, a member of the earliest branching mammalian lineage (Calabrese et al., 2017; Zhou et al., 2021) and four marsupials (Hazkani-Covo, 2022). In addition, we sampled organisms in clades where the genome size was relatively consistent (birds, reptiles, bats; full list in Supplementary Table S1). To minimize quality issues with poorly assembled GenBank genomes, we chose 21 RefSeq vertebrate genomes, and 6 GenBank genomes.

There is little correlation between genome size and either the amount of mitochondrial transfer (Figure 5) or the median length of the segments transferred (Supplementary Figure S4). The largest amount of transfer in our sample was seen with average sized (3 GB) genomes (*S. harrisii*, *T. cynocephalus*, and *A. flavipes*), the largest genomes in our sample (*A. mexicanum* and *N. forsteri*) had transfer amounts similar to the majority of organisms with genome sizes from 1.4 to 3 GB. Statistical analysis of the numt counts *versus* genome size in the 21 vertebrate RefSeq genomes, excluding genomes with no numts, revealed a weak (*p* < 0.02) Spearman rank correlation coefficient. For the same dataset, the Pearson correlation, which takes into account the magnitudes of both the genome sizes and numt counts, was not statistically significant.

### Numts cannot be detected in some vertebrates

As shown earlier (Figures 1, 2, 3, 5), the combination of sensitive TFASTX/MD40 protein:translated-DNA searches and the more complete whole-mitochondrial genome BLNGT searches produce the most comprehensive estimates of mtDNA-nuclear genome transfer. Because TFASTX/MD40 can find numt transfers that are not detected by other methods, it is possible that some of those transfers are alignment artifacts, e.g., false positives that do not represent genuine transfers. Evidence that there are very few false-positives, particularly with the most sensitive MD40BG merged count strategy, is provided by the vertebrate genome set. When we looked at numt hits under more stringent E()-values, using $<10^{-6}$ for TFASTX and $<10^{-10}$ for BLASTN, the median reduction in numts was ∼17 % (Supplementary Table S1). While most organisms in both rodent and vertebrate genome sets had between 100 and 1,000 numt transfers, three vertebrate genomes (*N. naja*, *T. rubripes* and X*. tropicalis*) appear to have no detectable mitochondrial nuclear transfers when measured with our most sensitive method (MD40BG; Figure 5). While each of those genomes showed some transfer with one or two of our methods, the alignments supporting those transfers had low expectation values, and we believe those alignments were false positives (T. *rubripes* TFASTX MD10 alignments had E()-values > 10^−4^, and the *X. tropicalis* MD10 alignments had E()-values ${>10}^{-5}$ ). The MD40BG strategy found 84 numts in *D. rerio*, but none of the numts were found by TFASTX/MD40 with E ()<10^−3^, and none of the DNA:DNA alignments had E ()-values $<10^{-8}$ , a generous threshold for DNA:DNA alignments. We do not believe the *D. rerio* alignments represent true nuclear transfers; 94% of the numts reported for *D. rerio* are lost under more stringent E()-values, TFASTX $<10^{-6}$ , BLASTN $<10^{-10}$ (Supplementary Table S1). With the large number of searches being analyzed, some false-positives are expected. However, while our hybrid TFASTX/BLASTN approach can detect more numt transfer than previous methods, there are still organisms with no apparent mitochondrial-nuclear transfer.

## Discussion

### MtDNA transfer is not more abundant in *Microtus* voles

MtDNA genomes contain a small fraction of the genes necessary for the function of the mitochondria; most mitochondrial proteins are coded by genes that have been transferred to the nucleus (Lang et al., 1999). There are also continued insertions of mtDNA genome fragments into the nuclear genome that are not expressed and endure as pseudogenes. Little is known about why some taxonomic lineages have infrequent or no mtDNA nuclear transfers, while others have transferred thousands of pieces of the mitochondrial genome to the nucleus. One of the initial goals of this study was to survey rodent genomes to test whether *Microtus* vole genomes harbored an increased number of numts that was driven by their plastic karyotype (Triant and DeWoody, 2008). If double-strand break repair mechanisms are involved in numt integration, as was postulated for primate genomes (Ricchetti et al., 2004), species that have undergone generations of chromosomal fusion events would seem good candidates for numt transfers.

We looked at mitochondrial nuclear transfer in 37 rodent genomes, using a variety of similarity searching strategies. Traditionally, mitochondrial nuclear transfers (numts) have been identified by looking for mitochondrial genomic fragments with BLASTN, and, more recently, SRA alignments. While BLASTN can identify many numt transfer events, DNA:DNA similarity searching is considerably less sensitive than protein:translated-DNA searching, so we also did searches using the mitochondrial encoded proteins. We used a scoring matrix that is optimal for about 90% identity (MD10) and a second that works best at about 65% identity (MD40). We also searched with the much more sensitive BLOSUM62 matrix, which targets alignments that are 30% identical (Pearson, 2013b), the default used by BLASTP and TBLASTN (Camacho et al., 2009). BLOSUM62 searches did not produce significantly more numts than MD40, but appeared to produce more false-positives (data not shown), so we used MD40 for our most sensitive searches. In addition to providing a greater evolutionary look-back time, protein:protein (or protein:translated-DNA) alignments have more consistent statistical properties; at a given expectation value, TFASTX alignments produce fewer false positives (Pearson, 2013a).

Despite using more sensitive search techniques, none of the vole species, particularly the three species of *Microtus* voles nor the two other Arvicoline voles in the rodent set (*A. amphibius* and *M. glareolus*), had an unusual number of numts, apart from *M. arvalis*. However, the *M. arvalis* genome is a lower quality GenBank genome assembled at the scaffold level. Because of the scaffold level assembly, we believe the *M. arvalis* numt count is an overestimate. We also included the muntjak deer, *Muntiacus muntjak*, which has a rapidly evolving mammalian karyotype (Wurster and Benirschke, 1967, 1970; Mudd et al., 2020). The muntjak genome is also a GenBank assembly but the genome is assembled at the chromosome level. *M. muntjak* has a numt count just above the third quartile in the rodent genome set. The rodent genomes sampled are relatively uniform in size (2.0 GB–3.7 GB; Supplementary Table S1) and the two species that did have a 10-fold higher amount of numts (*M. coypus* and *T. swinderianus*) have similar sized genomes (2.9 GB and 2.6 GB, respectively). Again, those high-numt genomes were GenBank genomes assembled at the scaffold level. We conclude that neither *Microtus* voles nor rodents are remarkable in their numt content and karyotypic plasticity does not seem to be driving mtDNA transfers.

### Similarity search strategies and numt estimates

We assessed seven search methods for finding numts including using the whole mtDNA genome with BLASTN. We note that the BLASTN -task option used is critical. BLASTN defaults to using “-task megablast”, which is fast but not very sensitive (targeting alignments that are more than 99% identical), and the alternative ‘‘-task blastn” option, uses more sensitive parameters and target sequences that are more than 80% identical. Searches with the complete mitochondrial genome and BLASTN “-task BLASTN” (BLNGT) found more than 90% of previously characterized human numts (Simone et al., 2011). Our results indicate that BLASTN with the “-task megablast” default found the fewest numts both using the protein-coding portions of the mtDNA genome, and when using the entire genome as a query (Figures 1, 2, 5). Many BLASTN users may not be aware of this lower sensitivity default. The low sensitivity BLASTN default is equivalent to aligning against the NCBI Sequence Read Archive (SRA). BLASTN “-task megablast” uses the same match/mismatch parameters as the short-read aligners required to align SRA reads. Just as “-task megablast” finds only half as many numts as BLASTN, SRA alignments would miss a similar number of numt insertions.

Protein:translated-DNA searches conducted with the protein-coding portion of the mtDNA genome using TFASTX found higher numbers of numts than BLASTN. Searching with the mtDNA genes also allowed us to ask whether some parts of the mtDNA genome (among the protein-coding genes) are more likely transferred than others, but we did not find any. Figure 4 shows that the mtDNA genes are uniformly represented in the nuclear genomes, after they are scaled by gene length. We also see more gene fragments transfers in GenBank genomes, suggesting that numt counts for GenBank genomes are inflated.

Searches with individual genes (TFASTX) and analyses of segments of complete BLASTN genomic searches (BLNGT) do not show preferential transfer of mitochondrial genome regions (Figure 4 and Supplementary Figure S3). To examine functional non-coding mtDNA genomic regions (e.g., the control region or the ribosomal RNAs 12S and 16S), we divided each mtDNA genome into 16 intervals (approximately 1,000 nt) and examined the median number of numts found in each interval in BLNGT searches (the entire mtDNA genome searched with BLASTN “-task blastn”, Supplementary Figure S3). As before, GenBank genomes produce higher counts, but counts across the intervals are relatively uniform. The intervals that contain the non-coding regions—12S rRNA (intervals 1,2), 16S rRNA (intervals 2,3)—are not overrepresented in their median numt counts. Likewise, we do not see differences in the control region (intervals 15,16), in contrast to other observations (Doynova et al., 2016; Calabrese et al., 2017).

Our most sensitive search strategy (MD40BG), Figures 1, 2, 5; Supplementary Figures S1, S2, S4) combines the sensitivity of TFASTX protein:translated-DNA searches with the MD40 protein scoring matrix, with the comprehensiveness of the BLASTN whole genome searches with the more sensitive “-task blastn” option. The scoring system used by TFASTX can detect alignments with more sequence changes, or lower percent identities, and finds more distant numts (Figure 3), while BLASTN “-task blastn” full-genome searches ensure that transfer from non-coding genes can be detected. The complementary effectiveness of the two approaches is shown in Figure 3, where percent identity is a proxy for evolutionary distance; older (more distant) numt transfers have lower identity. Although the rate of evolution on the mtDNA locus from which the numt originated can differ by location within the mtDNA genome and by organism, once the numt is transferred to the nuclear genome it is presumed to be non-functional and thus no longer under selection (Zischler et al., 1995; Bensasson et al., 2001). For both individual organisms (mouse, human) and the entire rodent and vertebrate genome sets, the TFASTX/MD40-only searches can identify lower identity (more distant) alignments, while the BLNGT-only alignments have higher identities. This is expected; protein:translated-DNA alignments can easily detect transfers with less than 50% identity, which are rarely found by DNA:DNA alignments (Pearson, 2019).

### Genome quality and numt detection

The observation that GenBank genomes tend to have higher apparent numbers of numt transfers than RefSeq genomes came as a surprise. However, the observation is consistent; we see it not only in rodent genomes (Figures 1, 3) but also in non-rodent vertebrates (Figure 5; Supplementary Figure S1) both when looking at the total number of numts and at longer, presumably “younger”, numt transfers. We see the same pattern when looking across the individual genes in the mitochondrial genome (Figure 4). RefSeq genomes are typically constructed from higher quality assemblies and go through additional gene-finding and annotation pipelines (O'Leary et al., 2016). In general, RefSeq genomes are better curated than GenBank genomes, and our results suggest that the higher quality RefSeq genome assemblies generate smaller numbers of numt alignments.

It is difficult to posit a biological explanation for the difference in the amount of numt transfer for RefSeq and GenBank genomes. A simpler explanation is that GenBank genomes are more preliminary and therefore, more likely to include duplicated or poorly assembled regions that will be merged in future genome releases, so that the numt count associated with those duplicated/unassembled regions will subsequently be reduced. While some GenBank genomes are assembled to the chromosome level, others can be to the scaffold or even contig level and have not been annotated or further curated by the NCBI (e.g., contaminants removed) (Benson et al., 2013). The four rodent species that show the highest number of numts regardless of alignment length (*C. sociabilis*, *M*. *arvalis*, *M. coypus*, *T. swinderianus*) (Figures 1, 3) are all GenBank genome assemblies at the scaffold assembly level with scaffold N50 ranging from 21 to 53 kb. Their numt counts are likely the results of a highly-fragmented genome. Before beginning a numt search, the quality of the genomes being used should be considered. It is also important to report the exact version of the assembly as improvements made between versions could potentially affect the number of numts discovered (Grau et al., 2020).

### Genome size and numt transfer

As with the rodents, there was not any clear biological pattern of numt transfer among vertebrate species (Figure 5). In particular, the species with the largest genomes (*A. mexicanum* (28.2 GB) and *N. forsteri* (34.6 GB), both GenBank genomes with assembled chromosomes, did not contain the largest number of numts. The large genome of *A. mexicanum* had fewer numts than other species with 10-fold smaller genomes (Figure 5). This lack of correlation is consistent with other findings (Puertas and González-Sánchez, 2020). However, Hazkani-Covo (2022) found a significant Spearman-rank correlation between genome size and numt content in vertebrates with genome sizes ranging from (0.38–5.3 GB). We performed a Spearman rank analysis on the 19 RefSeq vertebrates that contained numts (thus excluding *D. rerio*, *N. naja*, *T. rubripes*, and *X. tropicalis*), and found a weak correlation (*p* < 0.02). Significant correlations were not found with the Pearson measure, which we prefer, because it considers the magnitudes of the genome sizes and numt counts.

MtDNA insertions have been reported to be absent from fish or present in small numbers (Antunes and Ramos, 2005). We did not find any numts in the fish *T. rubripes* with our most sensitive search methods. Numts were found with only one or two methods in the *N. naja* and *X. tropicalis* genomes, and by the genomic BLNGT strategy in *D. rerio*. We believe these alignments are false positives, because they are not found with more sensitive methods and because they had marginal E()-values. When presented with a very small number of numts in a genome, attention should be paid to the alignment’s E()-value, as higher (less significant) values could indicate false positives rather than authentic transfers. In the genomes with hundreds to thousands of numts, most of the numts had extremely significant (E() < 10^−20^) expectation values.

We detect the largest numbers of numts both in count and length in *H. sapiens*, consistent with previous studies of human genomes (Jensen-Seaman et al., 2009; Lang et al., 2012; Dayama et al., 2014; Dayama et al., 2020; Popadin et al., 2022). Three marsupial genomes: *A. flavipes* and *S. harrisii*, both in the family Dasyuridae and the extinct *T. cynocephalus* (Figure 5), have high numt counts. The marsupial genomes are all close to 3 GB in size (range 3.1–3.2 GB) with two assembled to the chromosome level (*A. flavipes*—GenBank; *S. harrisii*—RefSeq) and the third at the scaffold assembly level (*T. cynocephalus*—GenBank). Two of the marsupials (*A. flavipes*, *S. harrisii)* were recently found to have high numt content with numbers similar to those in this study (*S. harrisii* 5,319 numts/2,054 kb this study, 3,450 numts/1,995 kb (Hazkani-Covo, 2022); *A. flavipes* 4,247 numts/876 kb, 2,813 numts/847 kb; *T. cynocephalus* 4,111 numts/742 kb, 435 numts/238 kb). While our numt lengths are similar for the two chromosome level assemblies, our combined search method found more numts. This may be the result of the protein-coding searches, which can find shorter, more diverged numts. For the lower quality scaffold level assemblies, both our counts and total lengths are higher, highlighting the challenges associated with searching more fragmented GenBank genomes. Likewise, our estimates for two high quality RefSeq bird genomes (*T. guttata*: 52 numts/28 kb total length; *G. gallus*: 37 numts/12 kb total length) are consistent with previous avian studies that have reported low numbers of mtDNA insertions [*T. guttata* 22 numts/10 kb total length; *G. gallus* 13 numts/9 kb total length; (Pereira and Baker, 2004; Liang et al., 2018)], although our more sensitive approach again finds more numts.

We used a range of numt detection techniques to estimate mitochondrial nuclear transfer and recommend a method that combines protein:translated-DNA (TFASTX) searches conducted with the protein-coding portion of the mtDNA genome with DNA:DNA (BLASTN “-task blastn”) searches with the entire mtDNA genome to capture numts from non-coding regions. We did not find any abundance of numts in the *Microtus* voles despite their rapid rates of chromosomal evolution. Mitochondrial transfer estimates from GenBank genomes should be viewed with caution, as highly fragmented genomes can artificially increase numt counts.

## Data Availability

The original contributions presented in the study are included in the article/Supplementary Material, further inquiries can be directed to the corresponding author.
