# Supplementary material for: Comparison of detection methods and genome quality when quantifying nuclear mitochondrial insertions in vertebrate genomes
Source: Front Genet. 2022 Nov 22;13:984513. doi: 10.3389/fgene.2022.984513 (PMC9723244; doi:10.3389/fgene.2022.984513)
Supplement: Supplementary file 3 [file DataSheet1.docx]

## Supplementary Figures


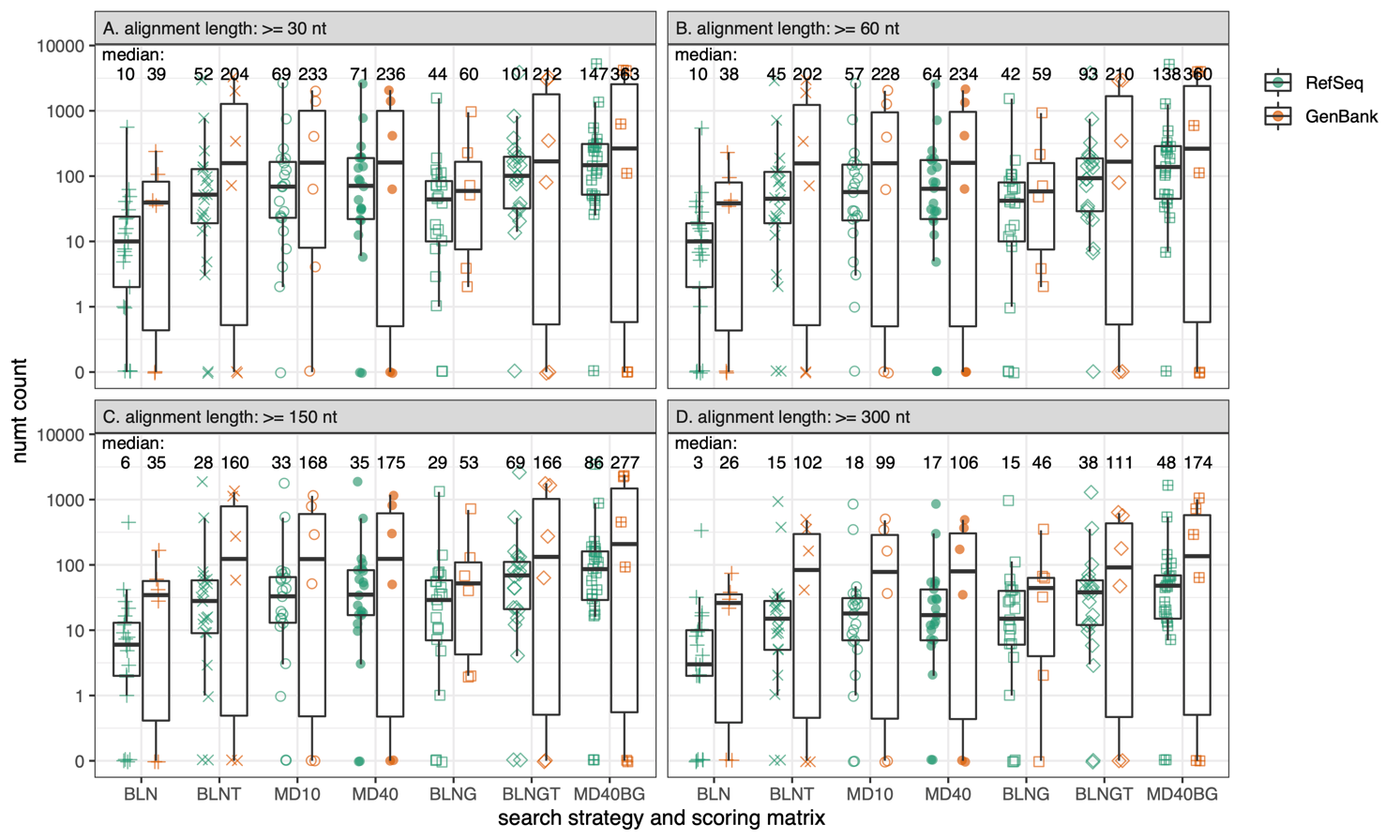


**Supplementary Figure 1.** Total numt counts for the vertebrate genome set for seven different similarity searches as a function of alignment length. The alignment length listed at the top of each panel is the minimum alignment length in nucleotides (nt). Median values are given for each type of similarity search with GenBank mtDNA genomes colored in orange and RefSeq mtDNA genomes colored in green. The symbols for the 7 different similarity searches run are the same as in **Figure 1**, and labeled on the x-axis.


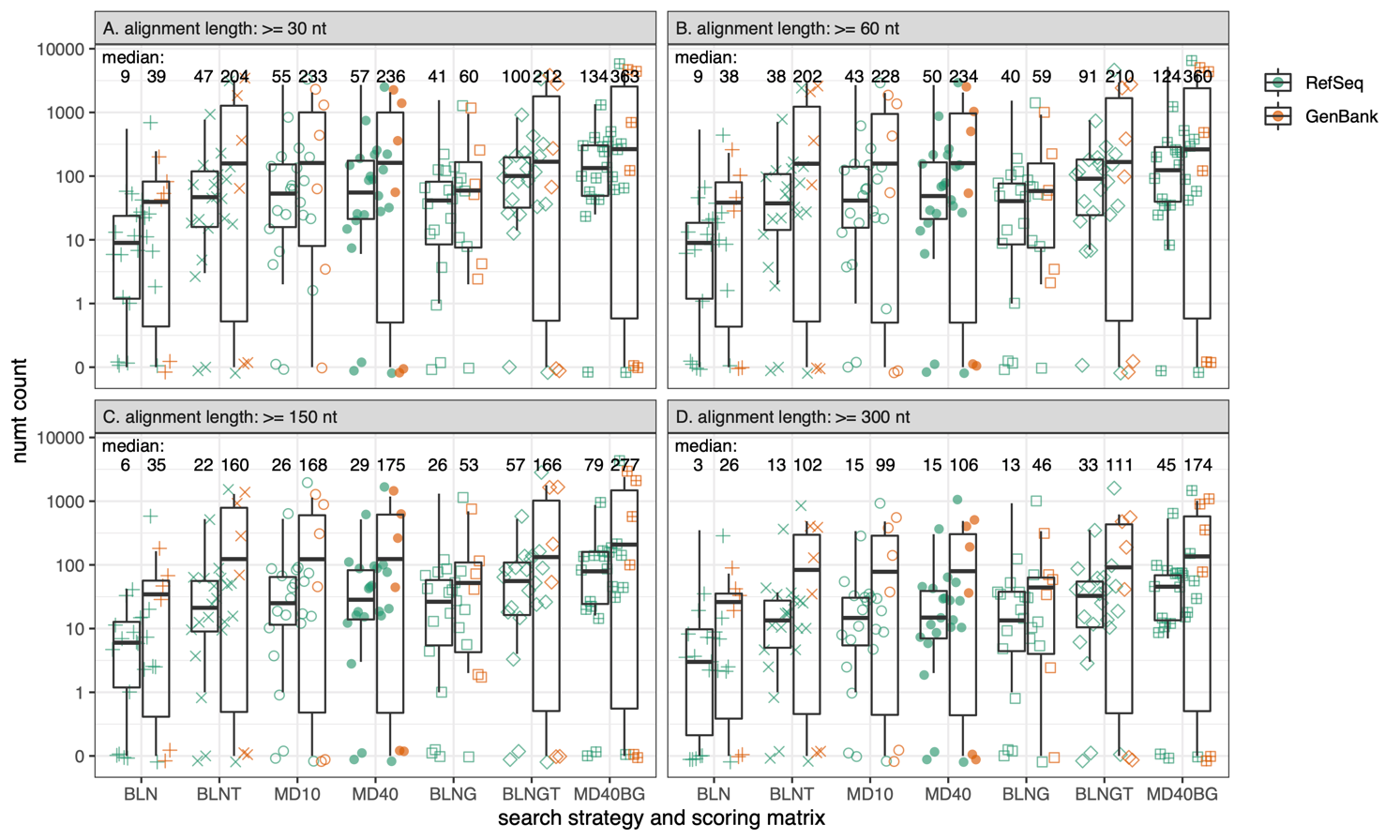


**Supplementary Figure 2.** Replotting the data from **Figure 2** excluding the four GenBank rodent genomes with the highest numt counts: *C.* sociabilis, *M. arvalis*, *M. coypus* and *T. swinderianus*. The symbols for the 7 different similarity searches run are the same as in **Figure 1**, and labeled on the x-axis.


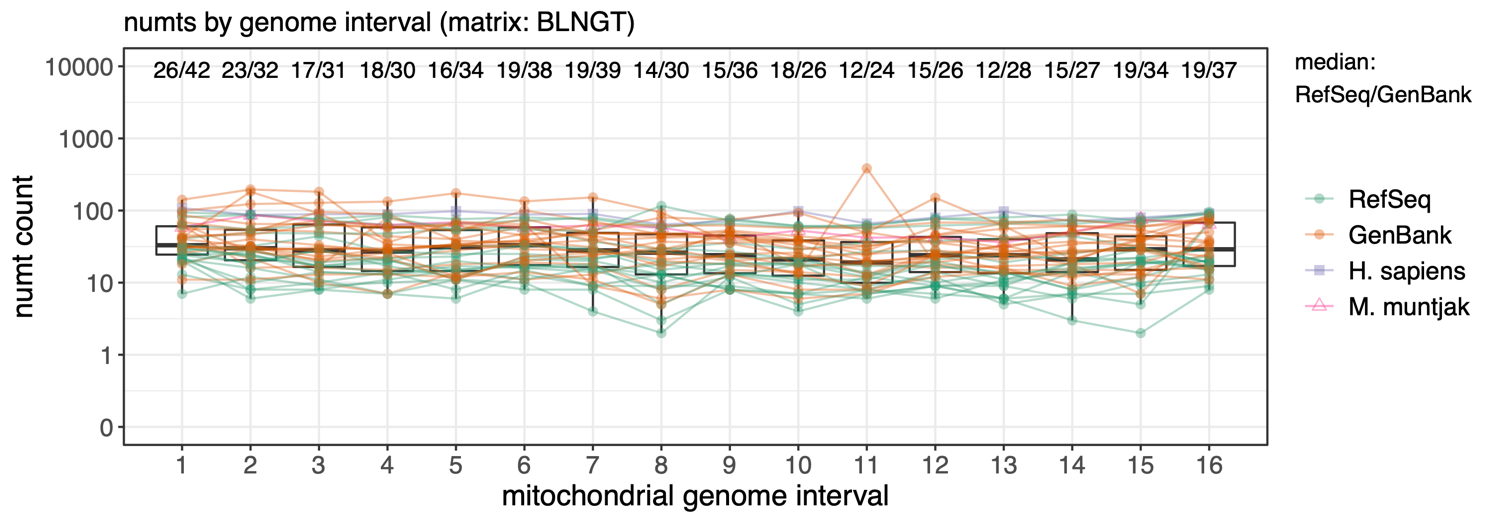


**Supplementary Figure 3.** Median number of numts found in each interval after dividing the mtDNA genome into 16 non-overlapping regions each approximately 1000 nt in size. Numt alignments were found using the combined search method done with whole mtDNA genomes using BLASTN with “-task blastn” (BLNGT). Median values for both RefSeq and GenBank are reported at the top of the figure. GenBank mtDNA genomes colored in orange and RefSeq mtDNA genomes colored in green.


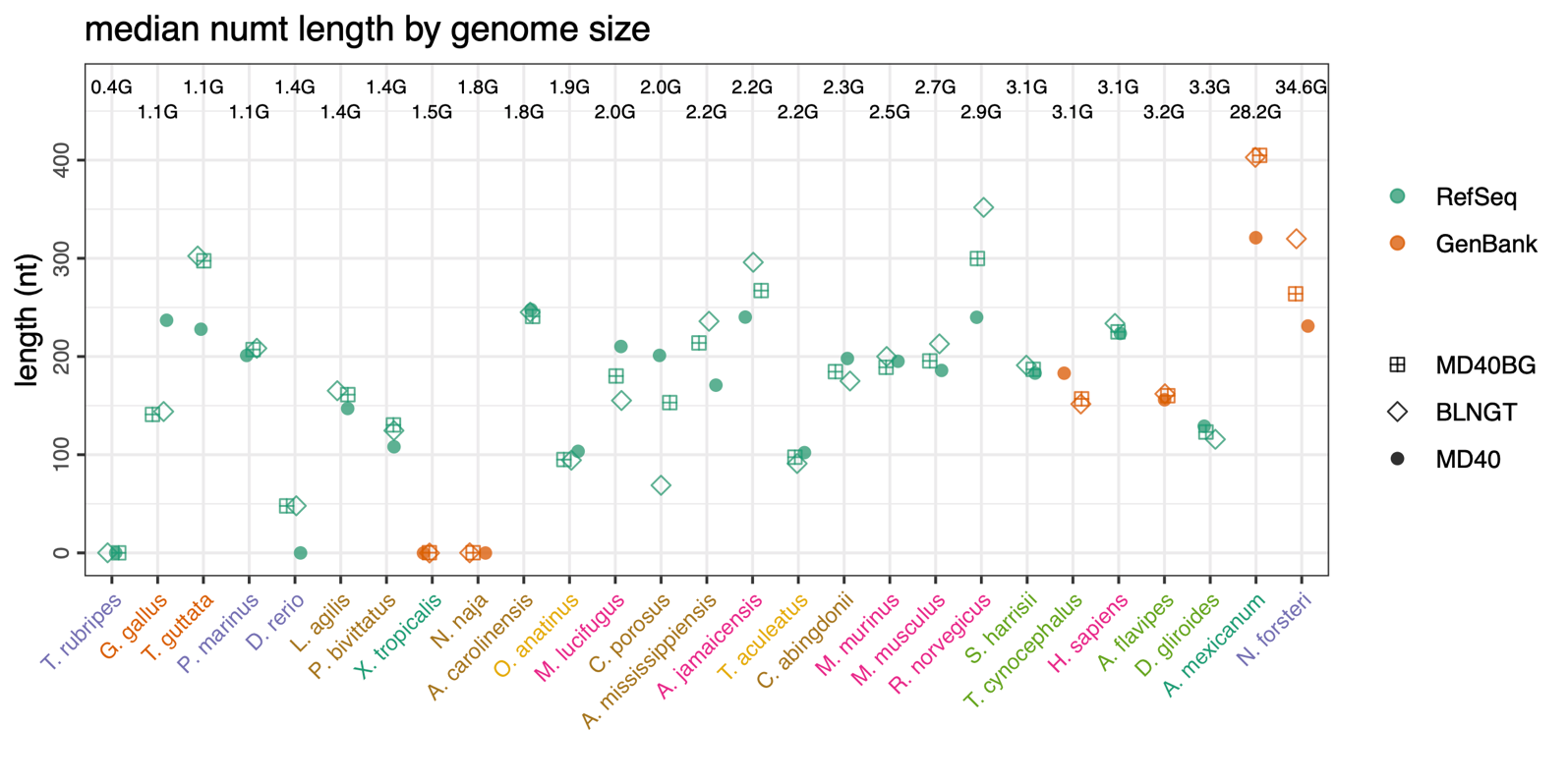


**Supplementary Figure 4.** Median numt length in nucleotides (nt) across vertebrate genomes. Genome sizes are listed at the top of the panel and species are listed at the bottom of the panel. Scientific names shown at the bottom of the panel are colored by vertebrate classes. GenBank mtDNA genomes colored in orange and RefSeq mtDNA genomes colored in green. Median numt counts for each species for the 3 different similarity searches run are shown as follows: 1) filled circle: coding sequence queries – TFASTX protein:translated-DNA searches with the MD40 protein scoring matrix (MD40); 2) diamond: whole mtDNA genome queries – BLASTN DNA:DNA with -task blastn option (BLNGT); 3) square with plus inside: combined numts found only with coding sequence queries TFASTX protein:translated-DNA with the MD40 scoring matrix, only those found with whole mtDNA genomes – BLASTN DNA:DNA that includes numts from non-coding portions of the genome and overlapping numts found with both (MD40BG). Scientific names are colored by class as in **Figure 5**.
